# Chromatographic Conditions as a Source of Analytical Variability in GC–MS Profiling of Essential Oils

**DOI:** 10.3390/molecules31162744

**Published:** 2026-08-07

**Authors:** Abdessamad Beraich, Daniela Batovska, Yousra Belbachir, Hasnae El Allaoui, Krastena Nikolova, Magdalena Szechyńska-Hebda, Anass Choukoud, Burak Dikici, Khadija Haboubi, Vincent Lequart, Patrick Martin, Abdelmonaem Talhaoui

**Affiliations:** 1 Laboratory of Environment and Applied Chemistry (LCAE), Team: Physical Chemistry of the Natural Resources and Processes, Department of Chemistry, Faculty of Sciences, Mohamed First University, Oujda 60000, Morocco; 2 Department of Mechanical Engineering, Faculty of Engineering, Ataturk University, 25240 Erzurum, Turkey; 3 Institute of Chemical Engineering, Bulgarian Academy of Sciences, Acad. G. Bonchev Str., Bl. 103, 1113 Sofia, Bulgaria; danibat@iche.bas.bg; 4 Laboratory of Engineering Sciences and Applications, National School of Applied Sciences of Al Hoceima, Abdelmalek Essâadi University, Al Hoceima 32003, Morocco; hasnae.elallaoui@etu.uae.ac.ma (H.E.A.);; 5 Department of Physics and Biophysics, Faculty of Pharmacy, Medical University of Varna, 84 Tzar Osvoboditel Blvd., 9000 Varna, Bulgaria; 6 Polish Academy of Sciences, W. Szafer Institute of Botany, 31-512 Krakow, Poland; m.szechynska-hebda@botany.pl; 7 Transformations and Agro-Resources Unit, Université d’Artois—UniLaSalle, ULR7519, F-62408 Béthune, Francepatrick.martin@univ-artois.fr (P.M.)

**Keywords:** GC–MS, essential oils, chromatographic conditions, non-polar columns, terpene profiling, analytical variability

## Abstract

The analytical characterization of essential oils (EOs) is often interpreted primarily in terms of biological variability, although analytical conditions may also affect compositional profiling and data comparability. In this study, the influence of chromatographic conditions on gas chromatography–mass spectrometry (GC–MS) profiling was investigated. Five EOs representing chemically diverse plant matrices, including *Pistacia lentiscus* (resin and stem) and leaf oils from *Salvia rosmarinus*, *Laurus nobilis*, and *Thymus vulgaris*, were analyzed using two capillary columns with different stationary phases: RTX-5MS (5% phenyl-substituted polysiloxane) and CP-Sil 5 CB (100% dimethylpolysiloxane). Comparative analysis revealed reproducible differences in chromatographic profiles and relative peak-area distribution, particularly within terpene-rich regions containing structurally related compounds. The most pronounced effects were observed for highly volatile monoterpenes, which were underrepresented under CP conditions. These findings demonstrate that chromatographic selectivity and column geometry may affect the semi-quantitative representation of EO profiles and contribute to variability in reported compositions. Overall, the results highlight the importance of carefully controlled analytical conditions in EO characterization.

## 1. Introduction

Essential oils (EOs) are complex mixtures of volatile secondary metabolites that play vital ecological roles in plants and serve as indispensable raw materials in the pharmaceutical, cosmetic, food, and fragrance industries [1,2,3,4,5]. Their chemical profiles dictate biological activity, sensory attributes, and commercial value; thus, accurate characterization is a prerequisite for quality control, authentication, and standardization [6,7,8].

A defining feature of EOs is their pronounced compositional variability, traditionally attributed to biological and environmental determinants, including plant species, organ specificity, phenological stage, and geographical origin [9,10,11,12,13]. These factors modulate biosynthetic pathways, leading to significant intra- and interspecific variation [14]. However, while EO composition is typically interpreted primarily through the lens of biology, the potential contribution of analytical methodology to semi-quantitative compositional differences is frequently underestimated [9,15]. Variations in chromatographic conditions may alter the apparent representation of volatile constituents and consequently influence phytochemical interpretation, chemometric classification, and the comparability of EO datasets. To ensure reliable characterization, the influence of instrumental conditions must be considered alongside biological factors.

The intrinsic complexity of EOs—characterized by numerous structurally related terpenes spanning wide concentration ranges—has driven the development of diverse gas chromatography (GC)-based methodologies aimed at enhancing separation efficiency and identification power. These include specialized sampling interfaces such as headspace and solid-phase microextraction (HS-GC–MS, HS-SPME–GC–MS), increased peak capacity through comprehensive two-dimensional gas chromatography (GC × GC–MS), and hyphenated detection systems including ion mobility spectrometry (GC–IMS), flame ionization detection (GC–FID), or olfactometry (GC–O). Additionally, enantioselective stationary phases and chemometric post-processing are frequently employed to resolve co-eluting isomers or handle high-dimensional data [6,7,8,16,17,18,19,20,21,22]. While complementary techniques such as nuclear magnetic resonance (NMR) spectroscopy or liquid chromatography–mass spectrometry (LC–MS) may assist in the structural elucidation of less volatile or thermolabile constituents [23,24,25], gas chromatography–mass spectrometry (GC–MS) remains the routine standard for EO profiling, owing to operational robustness, global accessibility, and the extensive depth of standardized mass spectral libraries [16,26].

Within the GC–MS framework, chromatographic selectivity—strongly influenced by stationary phase chemistry together with column dimensions and film characteristics—represents an important factor governing separation efficiency, particularly for isomeric monoterpenes and sesquiterpenes [18,27,28]. While non-polar to low-polarity 5% phenyl-substituted polysiloxane columns (e.g., HP-5MS, DB-5MS, RTX-5MS) and polar polyethylene glycol-based columns (e.g., DB-Wax, HP-Innowax) are widely adopted as complementary tools [6,18], chromatographic configuration may significantly influence compound detectability and semi-quantitative representation. Such shifts in the compositional representation may subsequently affect chemometric classification and the interpretation of biological or functional properties [27,28,29,30].

Despite these considerations, the influence of chromatographic selectivity on the semi-quantitative representation of chemically diverse essential oil matrices remains insufficiently examined, particularly regarding the comparability of results obtained on different non-polar phases. This issue is especially relevant for terpene-rich mixtures, where co-elution and peak compression may alter the apparent distribution of structurally related constituents and affect compositional interpretation. In this study, five EOs with distinct compositional characteristics were selected—including *Pistacia lentiscus* (resin and stem) and leaf oils from *Laurus nobilis*, *Salvia rosmarinus*, and *Thymus vulgaris*—to evaluate the influence of chromatographic conditions across varied terpene profiles. Analyses were performed using two capillary GC columns differing in stationary-phase chemistry, internal diameter, and film thickness—a 5% phenyl-substituted polysiloxane column (RTX-5MS) and a 100% dimethylpolysiloxane column (CP-Sil 5 CB)—to assess method-induced semi-quantitative variability. The aim of the study was to investigate how differences in chromatographic conditions may influence semi-quantitative compositional representation and the comparability of routine EO profiling data.

## 2. Results and Discussion

### 2.1. Chromatographic Differences Between RTX and CP Columns

Five EOs from different botanical sources and plant organs were selected to evaluate the effect of chromatographic conditions on GC–MS profiling: *P. lentiscus* resin (PL-R), *P. lentiscus* stem (PL-S), and leaf-derived oils from *S. rosmarinus* (SR-L), *L. nobilis* (LN-L), and *T. vulgaris* (TV-L). Analyses were performed using two GC capillary columns differing in stationary phase and geometry: RTX-5MS (5% diphenyl/95% dimethylpolysiloxane) and CP-Sil5 CB (100% dimethylpolysiloxane), hereafter referred to as RTX and CP, respectively.

All EOs showed clear chromatographic differences between RTX and CP (Figures S1–S5). These effects were most evident in regions containing multiple closely eluting constituents, where changes in column selectivity altered peak spacing and apparent peak separation [22,31,32]. Similar behavior has been reported for terpene-rich systems containing structurally related compounds with comparable volatility and polarity [33,34].

Because of the lower polarity of the CP column, several volatile constituents eluted at earlier retention times under the applied chromatographic conditions. Highly volatile monoterpenes such as α-pinene and camphene likely co-eluted with the solvent front before the 4 min acquisition start and were therefore underrepresented in the CP chromatograms. This effect was most evident in α-pinene-rich oils, including PL-R, PL-S, and SR-L (Figures S1–S3). Consequently, later-eluting minor compounds appeared relatively enriched in the CP profiles.

PL-R showed the strongest chromatographic distortion, particularly within the early monoterpene region (Figure S1). PL-S and SR-L displayed similar but less pronounced effects (Figures S2 and S3), mainly associated with reduced representation of early-eluting monoterpene hydrocarbons on the CP column. In contrast, LN-L and TV-L (Figures S4 and S5) showed less pronounced distortions because their dominant constituents, including 1,8-cineole, linalool, *p*-cymene, γ-terpinene, thymol, and carvacrol, eluted at later retention times and remained adequately represented in the CP chromatograms.

Inspection of the chromatograms additionally revealed matrix-dependent differences in peak crowding and signal distribution. TV-L and SR-L showed more compressed monoterpene-rich regions than LN-L, which exhibited a comparatively broader chromatographic distribution. CP chromatograms generally showed broader redistribution of oxygenated and later-eluting constituents, although local congestion remained evident in some highly dominant terpene-rich matrices. Similar improvements in the visibility of low-abundance compounds have been reported for optimized and multidimensional GC approaches applied to complex volatile systems [19,35].

The main matrix-dependent chromatographic characteristics observed for RTX and CP are summarized in Table 1.

### 2.2. Effect of Chromatographic Conditions on Terpene-Class Representation

To evaluate how chromatographic conditions influenced compositional interpretation, relative peak-area data obtained on RTX and CP were compared to evaluate column-dependent differences in compositional representation (Tables S1–S5). Although the principal constituents were generally detected under both analytical conditions, noticeable shifts in relative abundance were observed for several compounds, particularly among structurally related terpenoids [36].

For an integrated comparison across matrices, detected compounds were grouped into terpene classes: monoterpene hydrocarbons (MH), oxygenated monoterpenes (OxM), sesquiterpene hydrocarbons (SqH), and oxygenated sesquiterpenes (OxSq) (Table 2) [37].

Marked column-dependent differences in terpene class distribution were observed between RTX and CP. The most pronounced shifts occurred in PL-R, PL-S, and SR-L, where reduced representation of MH on CP was accompanied by increased relative contribution of OxM. LN-L showed the same general tendency, although the oil remained dominated by OxM under both chromatographic conditions. In contrast, TV-L displayed comparatively smaller changes in the MH/OxM balance, although the apparent contribution of SqH and OxSq increased on CP.

The observed shifts likely reflect differences in chromatographic resolution within crowded terpene-rich regions, where incomplete separation may alter relative peak-area representation [32,35]. Because GC–MS peak areas were not corrected using response factors, the reported percentages should be interpreted comparatively rather than as absolute quantitative values [30,31].

Oxygenated terpenoids appeared particularly affected by these shifts, likely because they frequently co-occur with structurally related hydrocarbon compounds and include numerous positional and stereoisomers with similar mass spectra [36,37]. Reduced detection of some early-eluting monoterpene hydrocarbons on CP may additionally reflect differences in chromatographic behavior affecting highly volatile constituents [22,30,31].

Overall, the results demonstrate that chromatographic conditions may introduce systematic semi-quantitative differences into EO compositional profiling. Transparent reporting of chromatographic parameters is therefore important for reliable inter-study comparison and EO standardization.

### 2.3. Comparative Visualization of Matrix-Dependent Chromatographic Behavior

**Figure 1:**
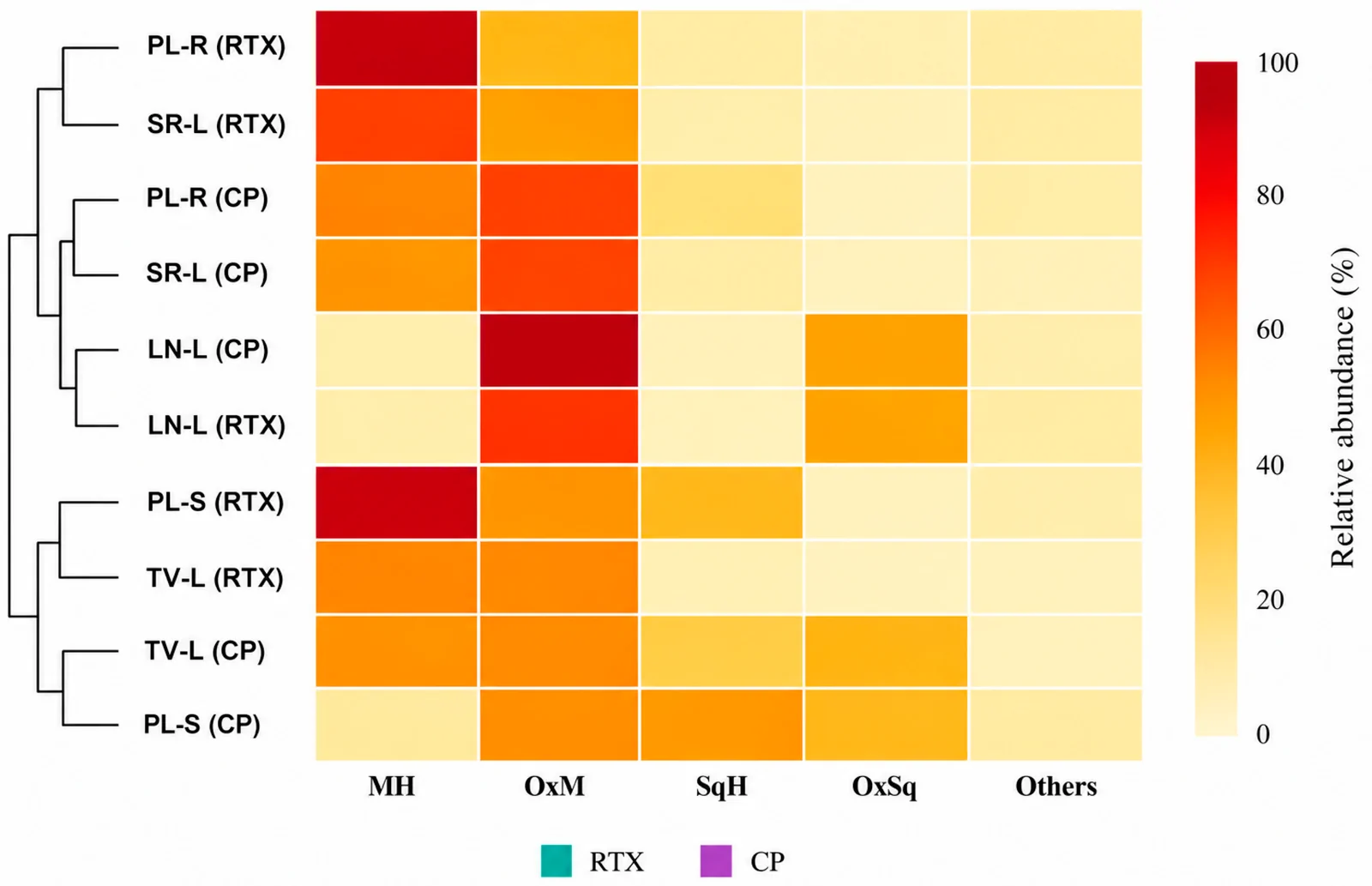
Clustered heatmap of terpene-class distribution (%) in essential oils analyzed using RTX and CP columns. Values were calculated from GC–MS peak areas and were uncorrected. Abbreviations: PL—*P. lentiscus*; LN—*L. nobilis*; SR—*S. rosmarinus*; TV—*T. vulgaris*; RTX—RTX-5MS column; CP—CP-Sil5 CB column; MH—monoterpene hydrocarbons; OxM—oxygenated monoterpenes; SqH—sesquiterpene hydrocarbons; OxSq—oxygenated sesquiterpenes.

Hierarchical clustering combined with heatmap analysis was used to visualize terpene-class distribution across the investigated chromatographic systems (Figure 1).

RTX datasets were generally associated with higher relative abundances of monoterpene hydrocarbons, whereas CP datasets showed increased representation of oxygenated fractions. Despite these shifts, RTX and CP profiles from the same essential oil remained more closely related to each other than to oils from different botanical sources.

### 2.4. Physicochemical Origin of Selectivity Differences Supported by Experimental Observations

The observed differences in peak resolution and compound detectability between RTX and CP likely reflect combined effects of stationary-phase chemistry and column geometry on terpene separation behavior. Both columns are based on polysiloxane matrices, but RTX contains approximately 5% phenyl substituents, whereas CP consists entirely of dimethylpolysiloxane [16,26,38]. These phenyl groups slightly increase polarizability and may enhance interactions with unsaturated and oxygenated terpenoids, despite the overall low polarity of both systems.

In capillary GC, separation is governed mainly by partitioning between the carrier gas and the stationary-phase film [18,22,38]. Retention depends primarily on dispersion forces, with weaker contributions from dipole-related and π-interactions [38]. Because terpenes differ in molecular geometry, unsaturation, and oxygenated functionality, even small variations in stationary-phase composition may influence their chromatographic behavior [38,39].

**Figure 2:**
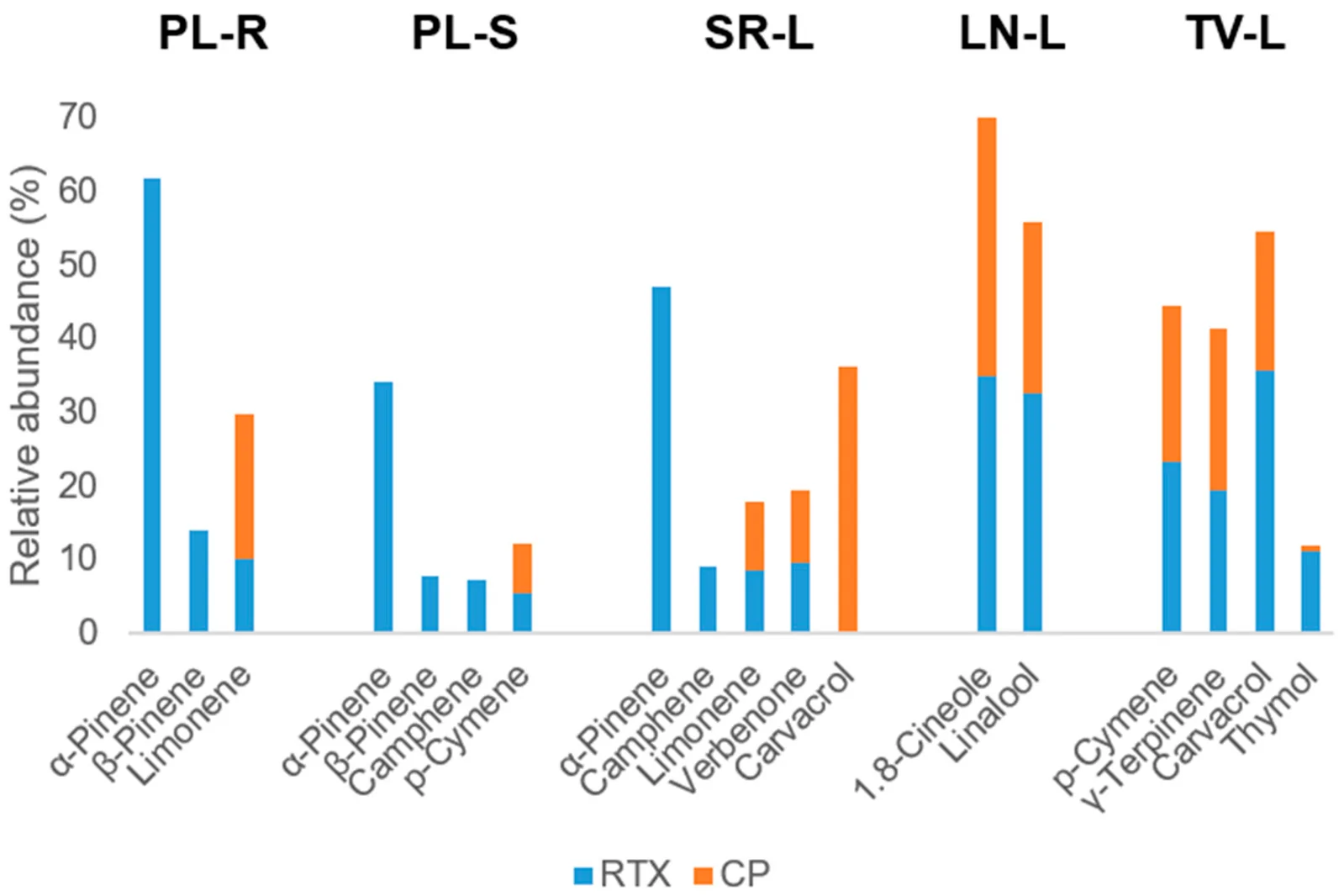
Column-dependent variation in the relative abundance (%) of major constituents in essential oils (PL-R, PL-S, SR-L, LN-L, and TV-L) analyzed using RTX and CP columns.

The practical consequences of these interactions were evident in the experimental data. In PL-R, RTX analysis showed apparent dominance of monoterpene hydrocarbons such as α-pinene, β-pinene, and limonene, whereas CP showed increased representation of limonene and several oxygenated derivatives, including *cis*-verbenol, *trans*-pinocarveol, and verbenone. Similar redistribution patterns were observed in PL-S, where oxygenated monoterpenes and sesquiterpenes became more prominent on CP. These differences likely reflect altered representation of closely eluting compounds under different chromatographic conditions rather than true compositional changes. Representative examples are shown in Figure 2.

Comparable effects were observed in leaf oils. In SR-L, CP showed higher apparent representation of oxygenated monoterpenes such as carvacrol and thymol, whereas RTX emphasized hydrocarbon constituents including α-pinene. In TV-L, both columns identified the thymol–carvacrol chemotype, although their relative proportions differed. LN-L showed smaller but measurable differences in major oxygenated constituents, including 1,8-cineole and linalool. Overall, these observations demonstrate that column selectivity may influence compositional representation across chemically diverse EOs.

Under the applied chromatographic conditions, the CP system appeared to favor separation patterns more strongly influenced by volatility-related interactions typical of dimethylpolysiloxane phases [38]. As a result, differences in peak separation altered the apparent representation of several lower-abundance and oxygenated constituents. The increased contribution of these compounds on CP therefore likely reflects chromatographic effects rather than true enrichment in the original EO. These effects are particularly relevant for structurally similar terpenoids and stereoisomers, where small differences in selectivity may substantially influence peak separation and relative peak-area representation [38,39].

Column geometry may further contribute to these differences. Variations in film thickness, internal diameter, and column dimensions influence phase ratio, mass-transfer kinetics, peak capacity, and resolution in densely populated chromatographic regions [40]. Consequently, the observed differences between RTX and CP likely reflect the combined influence of stationary-phase chemistry and column geometry [41].

The complementary behavior of the two chromatographic systems supports the use of orthogonal separation conditions for characterization of complex volatile mixtures [42]. Comparison of datasets obtained on columns with different selectivities may help identify regions where co-elution influences apparent compositional representation and semi-quantitative profiling. This is particularly relevant when reference standards or experimentally determined retention indices are unavailable.

## 3. Materials and Methods

### 3.1. Plant Material and Essential Oil Isolation

Plant materials originated from Morocco and were taxonomically authenticated by an expert at the collection site based on morphological characteristics. Material of *Pistacia lentiscus* was collected from a forest located approximately 35–40 km from the Faculty of Sciences in Oujda (34°31′04″ N, 1°50′35″ W). A voucher specimen has been deposited in the Herbarium of the University of Mohamed Premier (UMP) under voucher number UMPOM782. Leaves of *Laurus nobilis* and *Salvia rosmarinus* originated from the Triffa Plain (Berkane Province, 34°54′24″ N, 2°31′43″ W), whereas *Thymus vulgaris* was from the Taounate region (34°32′24″ N, 4°38′29″ W). Voucher specimens of *S. rosmarinus* and *L. nobilis* were deposited at the Center of Oriental Sciences and Technologies of Water and Environment (COSTE), Oujda, under voucher numbers CLM 26/26 and CLM 25/26, respectively. The *T. vulgaris* material was identified using standard botanical keys, and its voucher specimen (CLM24/26) was also deposited at COSTE, Oujda, Morocco.

EOs were obtained by single hydrodistillation using a Clevenger-type apparatus (Pyrex^®^, Corning Inc., Corning, NY, USA) [43]. *Pistacia lentiscus* resin (30 g) was processed in 500 mL of distilled water for 3 h, while stems (100 g) were treated under similar conditions using 800 mL of water. Dried leaves of *S. rosmarinus*, *L. nobilis*, and *T. vulgaris* (200 g each) were subjected to hydrodistillation for 3.5 h. The obtained EO samples were subsequently analyzed through multiple GC–MS injections under identical instrumental conditions.

The EOs were separated, dried over anhydrous sodium sulfate, and stored in sealed amber glass vials under nitrogen at 4 °C until analysis.

### 3.2. GC–MS Analysis

The chemical composition of five essential oils was analyzed using a Shimadzu gas chromatograph coupled to a QP2010 mass spectrometer (Shimadzu Corporation, Kyoto, Japan). Two capillary columns with different stationary-phase characteristics were employed: RTX-5MS (5% diphenyl/95% dimethylpolysiloxane; 30 m × 0.25 mm i.d.; 0.32 μm film thickness; Restek Corporation, Bellefonte, PA, USA) and CP-Sil5 CB (100% dimethylpolysiloxane; 30 m × 0.32 mm i.d.; 0.25 μm film thickness; Agilent Technologies, Santa Clara, CA, USA). It should be noted that the two columns also differed in internal diameter and film thickness, which may influence separation efficiency and detectability; therefore, the observed differences reflect the combined effects of stationary-phase chemistry and column geometry. The use of two chromatographic systems enabled comparative evaluation of chromatographic behavior and apparent compositional representation under different analytical conditions.

Helium (99.999%) was used as carrier gas at a constant flow rate of 1 mL min^−1^. The oven temperature was programmed from 50 °C (held 1 min) to 250 °C at 10 °C min^−1^. Samples (1 μL) were injected in split mode (split ratio 1:50). Mass spectra were recorded in electron ionization (EI) mode at 70 eV over an *m*/*z* range of 40–300.

Each EO sample was analyzed on both columns under identical instrumental conditions to evaluate chromatographic repeatability. Results are presented as mean relative peak area (%) ± SD from three replicate injections. Only constituents with values ≥1.0% were included in the comparative tables.

Compound annotation was based primarily on comparison of mass spectra with entries in the Wiley9/Nist11 (W9N11) mass spectral library (John Wiley & Sons, Hoboken, NJ, USA; National Institute of Standards and Technology, Gaithersburg, MD, USA) [44,45], together with evaluation of chromatographic behavior across both columns [46]. Compound assignments should be considered tentative due to the absence of experimentally determined retention indices and authentic reference standards.

### 3.3. Hierarchical Clustering and Heatmap Analysis

Semi-quantitative terpene class distribution data derived from uncorrected GC–MS peak areas were used for exploratory chemometric visualization. A clustered heatmap based on relative terpene class abundance (%) was generated to evaluate similarity patterns among the investigated EOs analyzed under different chromatographic conditions. Hierarchical clustering was performed using Euclidean distance and Ward’s linkage method based on terpene class distribution data [47]. Data visualization was performed using Python 3.11 (Python Software Foundation, Wilmington, DE, USA) and Microsoft Excel 365 (Microsoft Corp., Redmond, WA, USA).

## 4. Conclusions

This study demonstrates that chromatographic column selection influences the GC–MS profiling of essential oils. Comparative analysis using RTX and CP columns revealed systematic differences in peak resolution, relative peak-area distribution, and apparent representation of minor constituents across chemically diverse plant matrices. These effects reflected the combined influence of stationary-phase chemistry and column geometry on chromatographic behavior. The strongest differences were observed in highly volatile monoterpene-rich oils, where early-eluting constituents were underrepresented under CP conditions, resulting in increased apparent contribution of later-eluting and oxygenated compounds. Despite these systematic shifts, hierarchical clustering showed that RTX and CP datasets from the same essential oil remained more closely related to each other than to oils from different botanical sources. Overall, the results indicate that analytical conditions may contribute to variability in reported essential oil compositions alongside biological factors. The complementary behavior of RTX and CP supports the use of orthogonal chromatographic systems for more comprehensive interpretation of complex volatile mixtures, particularly when reference standards and experimentally determined retention indices are unavailable. Carefully controlled and transparently reported analytical conditions are therefore important for improving inter-study comparability and EO standardization.

## Figures and Tables

**Table 1:** Comparative overview of matrix-dependent chromatographic behavior observed for the investigated essential oils analyzed on RTX and CP columns.

Sample	Column	Dominant Behavior	Chromatographic Quality	Main Implication
PL-R	RTX	Early monoterpene compression	Stable	Signal dominance
PL-R	CP	Redistributed crowded profile	Congested	Expanded oxygenated representation
PL-S	RTX	Broad terpene distribution	Stable	Dispersed matrix
PL-S	CP	Expanded profile	Efficient	Favorable CP selectivity
SR-L	RTX	Compressed monoterpene region	Moderate	RT crowding
SR-L	CP	Structured oxygenated profile	Excellent	Optimal separation
LN-L	RTX	Distributed profile	Stable	Balanced matrix
LN-L	CP	Dense distributed profile	Good	Transitional behavior
TV-L	RTX	Severe compression	Overloaded	Dominance effects
TV-L	CP	Oxygenated congestion regions	Locally unstable	Selectivity saturation

**Table 2:** Distribution of terpene classes (%) in essential oils obtained on RTX-5MS and CP-Sil5 CB capillary columns.

Essential Oil	Column	MH (%) ±SD	OxM (%) ±SD	SqH (%) ±SD	OxSq (%) ±SD	Others (%) ±SD	Total (%)
*P. lentiscus* resin	RTX	95.00 ± 0.72	4.27 ± 0.04	0.16 ± 0.04	0	0.57 ± 0.006	100
	CP	33.14 ± 0.33	60.03 ± 0.62	3.75 ± 0.09	0.68 ± 0.006	2.40 ± 0.003	100
*P. lentiscus* stem	RTX	71.46 ± 0.69	15.69 ± 0.15	10.73 ± 0.10	0	2.12 ± 0.002	100
	CP	7.13 ± 0.06	44.33 ± 0.44	36.27 ± 0.40	5.88 ± 0.007	6.42 ± 0.007	100
*S. rosmarinus* leaf	RTX	71.97 ± 0.70	27.37 ± 0.27	0	0	0.66 ± 0.001	100
	CP	20.73 ± 0.21	79.27 ± 0.80	0	0	0	100
*L. nobilis* leaf	RTX	9.88 ± 0.008	75.69 ± 0.77	2.00 ± 0.005	4.68 ± 0.04	7.75 ± 0.008	100
	CP	0.75 ± 0.07	90.86 ± 0.86	0.34 ± 0.003	8.05 ± 0.08	0	100
*T. vulgaris* leaf	RTX	50.36 ± 0.53	48.73 ± 0.04	0.84 ± 0.83	0	0.07 ± 0.00001	100
	CP	43.06 ± 0.34	43.90 ± 0.50	6.78 ± 0.06	5.44 ± 0.03	0.82 ± 0.008	100

Abbreviations: MH—monoterpene hydrocarbons; OxM—oxygenated monoterpenes; SqH—sesquiterpene hydrocarbons; OxSq—oxygenated sesquiterpenes; Others—non-terpenoid volatiles. Relative percentages were calculated from uncorrected GC–MS peak areas without correction factors and are presented for comparative purposes.

## Data Availability

The original contributions presented in this study are included in the article/Supplementary Materials. Further inquiries can be directed to the corresponding authors.

## Supplementary Materials

The following supporting information can be downloaded at: https://www.mdpi.com/article/10.3390/molecules31162744/s1, Table S1: GC–MS analysis of *Pistacia lentiscus* resin essential oil using RTX-5MS and CP-Sil5 CB capillary columns; Table S2: GC–MS analysis of *Pistacia lentiscus* stem essential oil using RTX-5MS and CP-Sil5 CB capillary columns; Table S3: GC–MS analysis of *Salvia rosmarinus* leaf essential oil using RTX-5MS and CP-Sil5 CB capillary columns; Table S4: GC–MS chemical composition of *Laurus nobilis* leaf essential oil obtained using RTX-5MS and CP-Sil5 CB capillary columns; Table S5: GC-MS analysis of *Thymus vulgaris* leaf essential oil using RTX-5MS and CP-Sil5 CB capillary columns; Figure S1: GC–MS chromatograms of *Pistacia lentiscus* resin essential oil obtained on RTX and CP capillary columns; Figure S2: GC–MS chromatograms of *Pistacia lentiscus* stem essential oil obtained on RTX and CP capillary columns; Figure S3: GC–MS chromatograms of *Salvia rosmarinus* leaf essential oil obtained on RTX and CP capillary columns; Figure S4: GC–MS chromatograms of *Laurus nobilis* lead essential oil obtained on RTX and CP capillary columns; Figure S5: GC–MS chromatograms of *Thymus vulgaris* leaf essential oil obtained on RTX and CP capillary columns.

## Abbreviations

The following abbreviations are used in this manuscript:

CP	CP-Sil5 CB capillary column
EO(s)	Essential oil(s)
GC	Gas chromatography
GC–FID	Gas chromatography–flame ionization detection
GC–IMS	Gas chromatography–ion mobility spectrometry
GC–MS	Gas chromatography–mass spectrometry
GC × GC–MS	Comprehensive two-dimensional gas chromatography–mass spectrometry
GC–O	Gas chromatography–olfactometry
HCA	Hierarchical cluster analysis
HS-GC–MS	Headspace gas chromatography–mass spectrometry
HS-SPME	Headspace solid-phase microextraction
LC–MS	Liquid chromatography–mass spectrometry
LN-L	*Laurus nobilis* leaf essential oil
MH	Monoterpene hydrocarbons
NMR	Nuclear magnetic resonance
OxM	Oxygenated monoterpenes
OxSq	Oxygenated sesquiterpenes
PCA	Principal component analysis
PL-R	*Pistacia lentiscus* resin essential oil
PL-S	*P. lentiscus* stem essential oil
SR-L	*Salvia rosmarinus* leaf essential oil
RTX	RTX-5MS capillary column
SqH	Sesquiterpene hydrocarbons
TV-L	*Thymus vulgaris* leaf essential oil
