# Supplementary material for: Chromatographic Conditions as a Source of Analytical Variability in GC–MS Profiling of Essential Oils
Source: Molecules. 2026 Aug 7;31(16):2744. doi: 10.3390/molecules31162744 (PMC13515688; doi:10.3390/molecules31162744)
Supplement: Supplementary file 1 [file molecules-31-02744-s001.zip › molecules-4329544-supplementary.pdf]

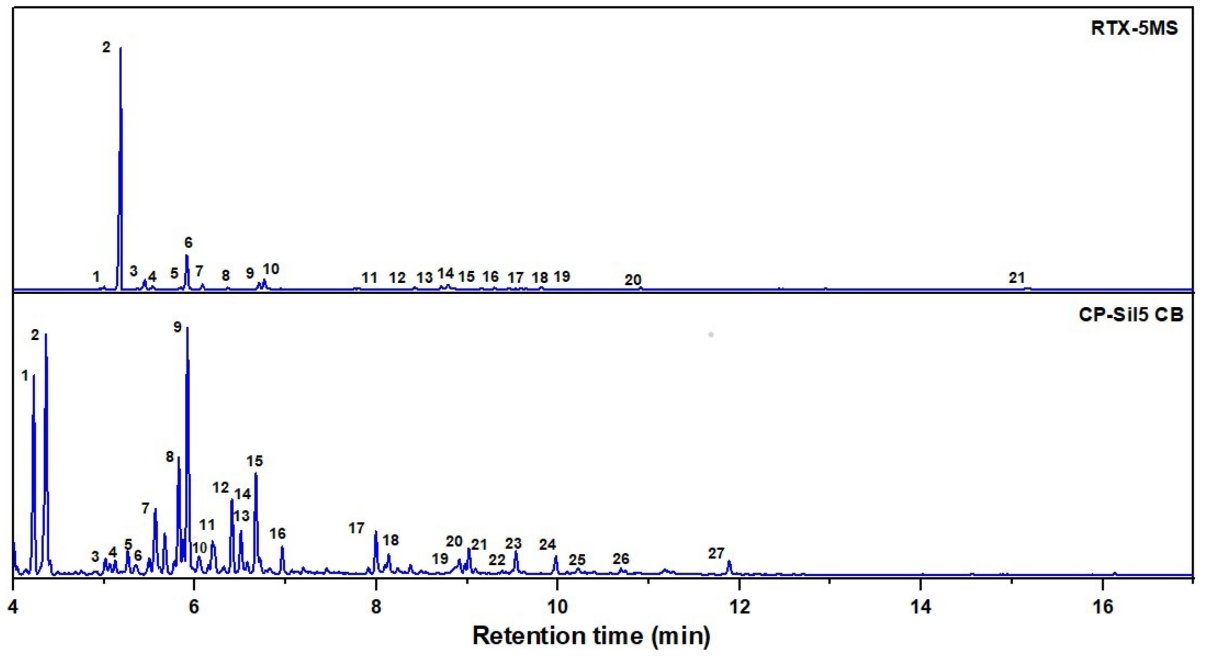

Figure S1. GC–MS chromatograms of *Pistacia lentiscus* resin essential oil obtained on RTX and CP capillary columns.

Table S1. GC–MS analysis of *Pistacia lentiscus* resin essential oil using RTX-5MS and CP-Sil5 CB capillary columns.

Component	RTX-5MS			CP-Sil5 CB		
	Peak	tR (min)	Area (%) ±SD	Peak	tR (min)	Area (%) ±SD
α-Pinene	2	5.2	61.82±0.123		n.d.*	n.d.
Camphene	3	5.4	4.27±0.009		n.d.	n.d.
β-Pinene	6	5.9	13.89±0.028		n.d.	n.d.
p-Cymene	9	6.7	2.79±0.005	1	4.2	13.28±0.027
Limonene	10	6.8	10.00±0.020	2	4.4	19.86±0.039
Linalool		n.d.	n.d.		5.3	1.74±0.009
α-Campholenal		8.4	0.18±0.0003	6	5.6	4.94±0.016
trans-Pinocarveol		8.7	0.77±0.002	8	5.8	7.77±0.033
cis-Verbenol		n.d.	n.d.	9	5.9	16.45±0.003
Pinocarvone		n.d.	n.d.	10	6.0	1.54±0.007
α-Phellandren-8-ol		n.d.	n.d.	11	6.2	3.42±0.012
Terpinen-4-ol		9.3	0.47±0.001	12	6.4	6.02±0.003
Myrtenal		n.d.	n.d.	13	6.5	1.44±0.015
cis-Carveol	14	8.8	1.51±0.003		n.d.	n.d.
Verbenone		n.d.	n.d.	14	6.7	7.49±0.003
trans-Carveol		n.d.	n.d.	15	7.0	1.71±0.009
Bornyl acetate		10.9	0.28±0.001	16	8.0	4.37±0.003
trans-*p*-Menth-2,8-dienol		n.d.	n.d.	19	9.0	1.29±0.003
Caryophyllene		12.9	0.16±0.0003	21	10.0	1.61±0.003

*n.d.— not detected under the applied analytical conditions.; tR—retention time (min); Area—relative peak area (%).

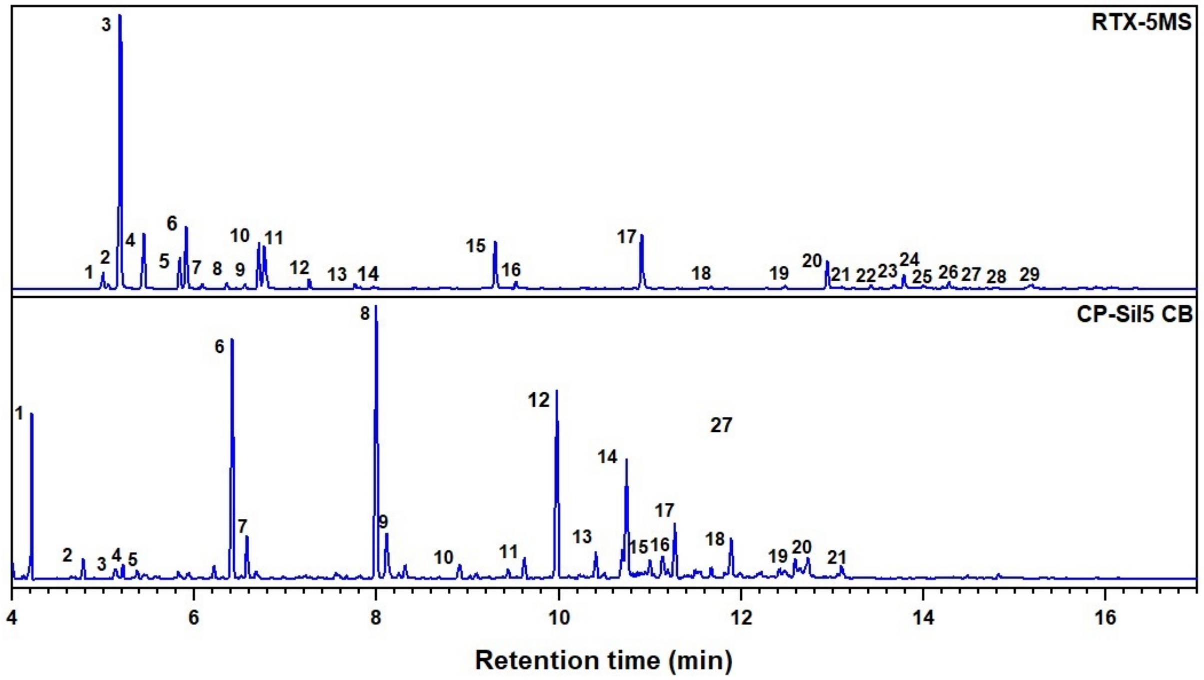

Figure S2. GC–MS chromatograms of *Pistacia lentiscus* stem essential oil obtained on RTX and CP capillary columns.

Table S2. GC–MS analysis of *Pistacia lentiscus* stem essential oil using RTX-5MS and CP-Sil5 CB capillary columns.

Component	RTX-5MS			CP-Sil5 CB		
	**Peak**	**tR (min)**	**Area (%) ±SD**	**Peak**	**tR (min)**	**Area (%) ±SD**
Tricyclene	2	5.0	2.05±0.006		n.d.*	n.d.
α-Pinene	3	5.2	34.23±0.103		n.d.	n.d.
Camphene	4	5.4	7.34±0.022		n.d.	n.d.
Sabinene	5	5.8	3.81±0.011		n.d.	n.d.
β-Pinene	6	5.9	7.83±0.023		n.d.	n.d.
p-Cymene	10	6.7	5.45±0.016	1	4.2	6.72±0.020
Limonene	11	6.8	6.69±0.020		n.d.	n.d.
Δ-3-Carene	12	7.3	1.19±0.004		5.2	0.41±0.001
Terpinen-4-ol	15	9.3	6.20±0.019	6	6.4	19.01±0.057
α-Terpineol	16	9.5	1.07±0.003	7	6.6	2.28±0.007
Bornyl acetate	17	10.9	7.67±0.023	8	8.0	22.13±0.066
Germacrene B		n.d.	n.d.	11	9.6	1.01±0.003
Caryophyllene		12.9	3.85±0.012	12	10.0	15.95±0.048
cis-α-Bisabolene		n.d.	n.d.	13	10.4	2.08±0.006
α-Cubebene		n.d.	n.d.	14	10.7	10.43±0.031
Caryophyllene oxide		n.d.	n.d.	18	11.9	4.20±0.013
Isoledene	24	13.8	2.27±0.007		n.d.	n.d.
δ-Cadinene	27	14.3	1.06±0.003	17	11.3	4.81±0.014

*n.d.— not detected under the applied analytical conditions.; tR—retention time (min); Area—relative peak area (%).

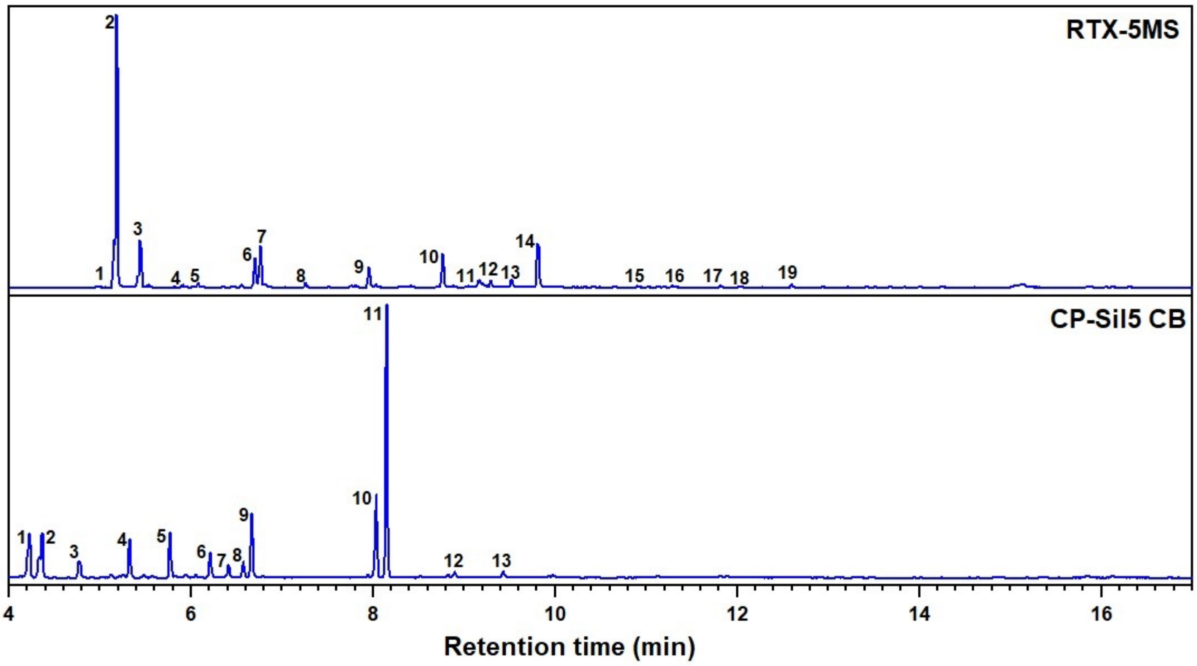

Figure S3. GC–MS chromatograms of *Salvia rosmarinus* leaf essential oil obtained on RTX and CP capillary columns.

Table S3. GC–MS analysis of *Salvia rosmarinus* leaf essential oil using RTX-5MS and CP-Sil5 CB capillary columns.

Component	RTX-5MS			CP-Sil5 CB		
	Peak	tR (min)	Area (%) ±SD	Peak	tR (min)	Area (%) ±SD
α-Pinene	2	5.2	47.05±0.094		n.d.*	n.d.
Camphene	3	5.4	9.15±0.018		n.d.	n.d.
p-Cymene	6	6.7	5.64±0.011	1	4.2	8.52±0.017
Limonene	7	6.8	8.65±0.017	2	4.4	9.17±0.018
γ-Terpinene	8	7.3	0.63±0.001	3	4.8	3.04±0.006
Linalool	9	8.0	3.98±0.008	4	5.3	5.74±0.011
Camphor	10	8.8	6.52±0.013	5	5.8	6.29±0.013
Borneol	11	9.2	2.96±0.006	6	6.2	3.68±0.007
Terpinen-4-ol	12	9.3	1.65±0.003	8	6.4	2.11±0.004
α-Terpineol	13	9.5	1.94±0.004	9	6.6	2.55±0.005
Verbenone	14	9.8	9.59±0.019	10	6.7	9.87±0.020
Carvacrol		n.d.	n.d.	11	8.1	36.24±0.072
α-Terpinyl acetate		n.d.	n.d.	12	8.9	0.51±0.001
Thymol	16	11.3	0.37±0.001	13	8.0	11.68±0.023

*n.d.— not detected under the applied analytical conditions.; tR—retention time (min); Area—relative peak area (%).

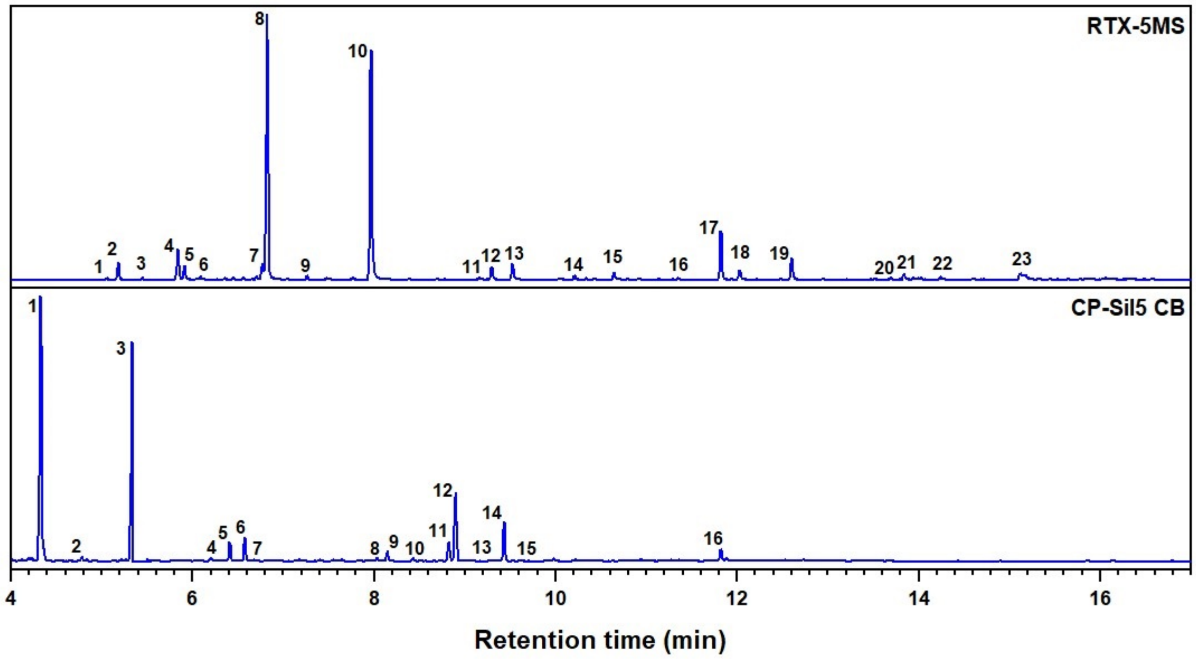

Figure S4. GC–MS chromatograms of *Laurus nobilis* lead essential oil obtained on RTX and CP capillary columns.

Table S4. GC–MS chemical composition of *Laurus nobilis* leaf essential oil obtained using RTX-5MS and CP-Sil5 CB capillary columns.

Component	RTX-5MS			CP-Sil5 CB		
	Peak	tR (min)	Area (%) ±SD	Peak	tR (min)	Area (%) ±SD
α-Pinene	2	5.2	2.24±0.004		n.d.*	n.d.
Sabinene	4	5.8	3.96±0.008		n.d.	n.d.
β-Pinene	6	5.9	2.02±0.006		n.d.	n.d.
1,8-Cineole	8	6.8	34.95±0.104	1	4.3	41.71±0.125
Linalool	10	8.0	32.51±0.097	3	5.3	23.27±0.070
Terpinen-4-ol	12	9.3	2.04±0.061	5	6.4	3.12±0.009
α-Terpineol	13	9.5	2.56±0.008	6	6.6	3.67±0.011
Carvacrol		n.d.	n.d.	9	8.2	1.62±0.005
Geranial	15	10.6	1.06±0.003		n.d.	n.d.
Δ-2-Carene	17	11.8	7.04±0.021		n.d.	n.d.
Eugenol	18	12.0	1.84±0.006	11	8.8	3.24±0.008
trans-β-Terpinyl butanoate		n.d.	n.d.	12	8.9	11.03±0.033
Isohomogenol	19	12.6	3.36±0.010	14	9.4	5.76±0.017
Spathulenol	23	15.1	1.32±0.004		n.d.	n.d
Isoaromadendrene epoxide		n.d.	n.d.	16	11.8	2.29. ±*0.007*

*n.d.—not detected under the applied analytical conditions.; tR—retention time (min); Area—relative peak area (%).

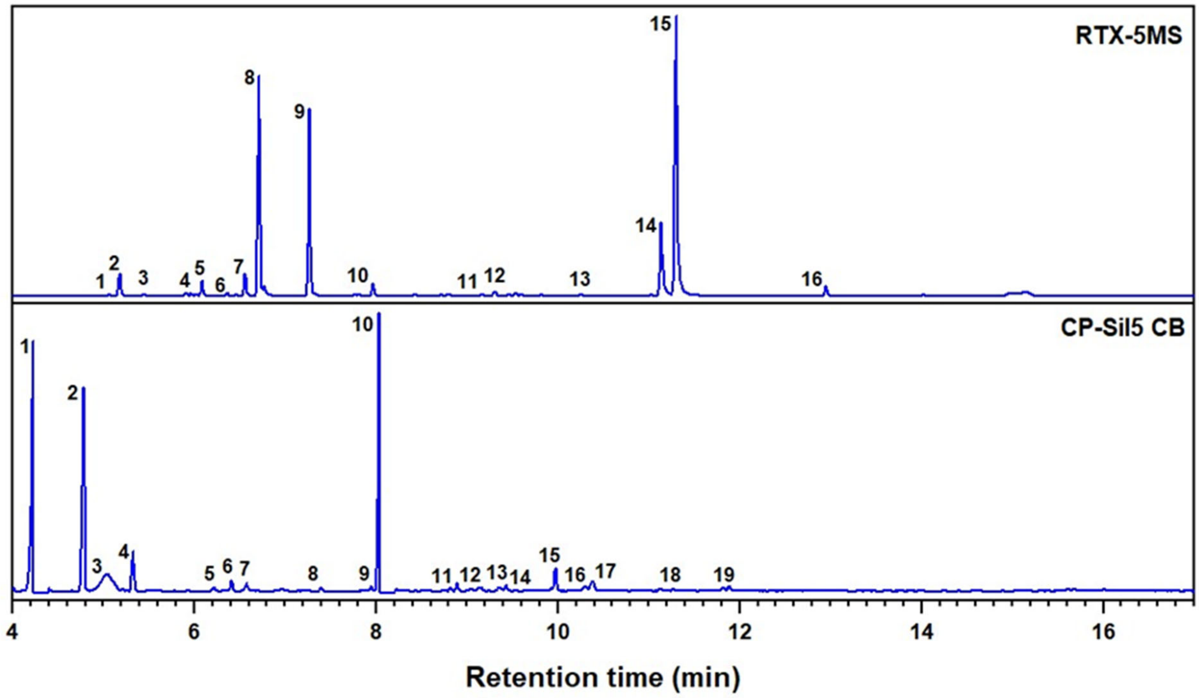

Figure S5. GC–MS chromatograms of *Thymus vulgaris* leaf essential oil obtained on RTX and CP capillary columns.

Table S5. GC-MS analysis of *Thymus vulgaris* leaf essential oil using RTX-5MS and CP-Sil5 CB capillary columns.

Component	RTX-5MS			CP-Sil5 CB		
	Peak	tR (min)	Area (%) ±SD	Peak	tR (min)	Area (%) ±SD
α-Pinene	2	5.2	2.88±0.009		n.d.*	n.d.
β-Myrcene	5	6.1	1.70±0.005		n.d.	n.d.
α-Phellandrene	6	6.4	0.13±0.004		n.d.	n.d.
α-Terpinene	7	6.6	2.47±0.007		n.d.	n.d.
p-Cymene	8	6.7	23.29±0.069	1	4.2	21.14±0.063
γ-Terpinene	9	7.3	19.46±0.58	2	4.8	21.92±0.066
Verbenol		n.d.	n.d.	3	5.0	11.51±0.035
Linalool	10	8.0	1.48±0.004	4	5.3	4.7±0.014
Borneol		n.d.	n.d.	5	6.2	1.1±0.003
Terpinen-4-ol		n.d.	n.d.	6	6.4	2.52±0.008
α-Terpineol	11	9.5	0.22±0.006	7	6.6	1.96±0.002
Thymol	13	11.1	11.05±0.033	9	7.9	0.8±0.056
Carvacrol	14	11.3	35.7±0.107	10	8.0	18.76±0.004
α-Terpinyl acetate		n.d.	n.d.	11	8.9	1.45±0.004
β-Bisabolene		n.d.	n.d.	12	9.1	1.43±0.004
Isoledene		n.d.	n.d.	13	9.4	1.4±0.003
Eugenol		n.d.	n.d.	14	9.4	1.1±0.009
Caryophyllene	15	13.0	0.84±0.003	15	10.0	3.24±0.004
Spathulenol		n.d.	n.d.	16	10.3	1.79±0.003
Caryophyllene oxide		n.d.	n.d.	17	10.4	2.59±0.002
Isoaromadendrene epoxide		n.d.	n.d.	19	11.9	1.06±0.004

*n.d.— not detected under the applied analytical conditions.; tR—retention time (min); Area—relative peak area (%).
